# Supplementary figures and images for: A modified GC-specific MAKER gene annotation method reveals improved and novel gene predictions of high and low GC content in Oryza sativa
Source: BMC Bioinformatics. 2017 Nov 25;18:522. doi: 10.1186/s12859-017-1942-z (PMC5702205; doi:10.1186/s12859-017-1942-z)

"Hypothetical"  
Genes  
(1324)

GC-Specific Novel  
Low GC  
(369)

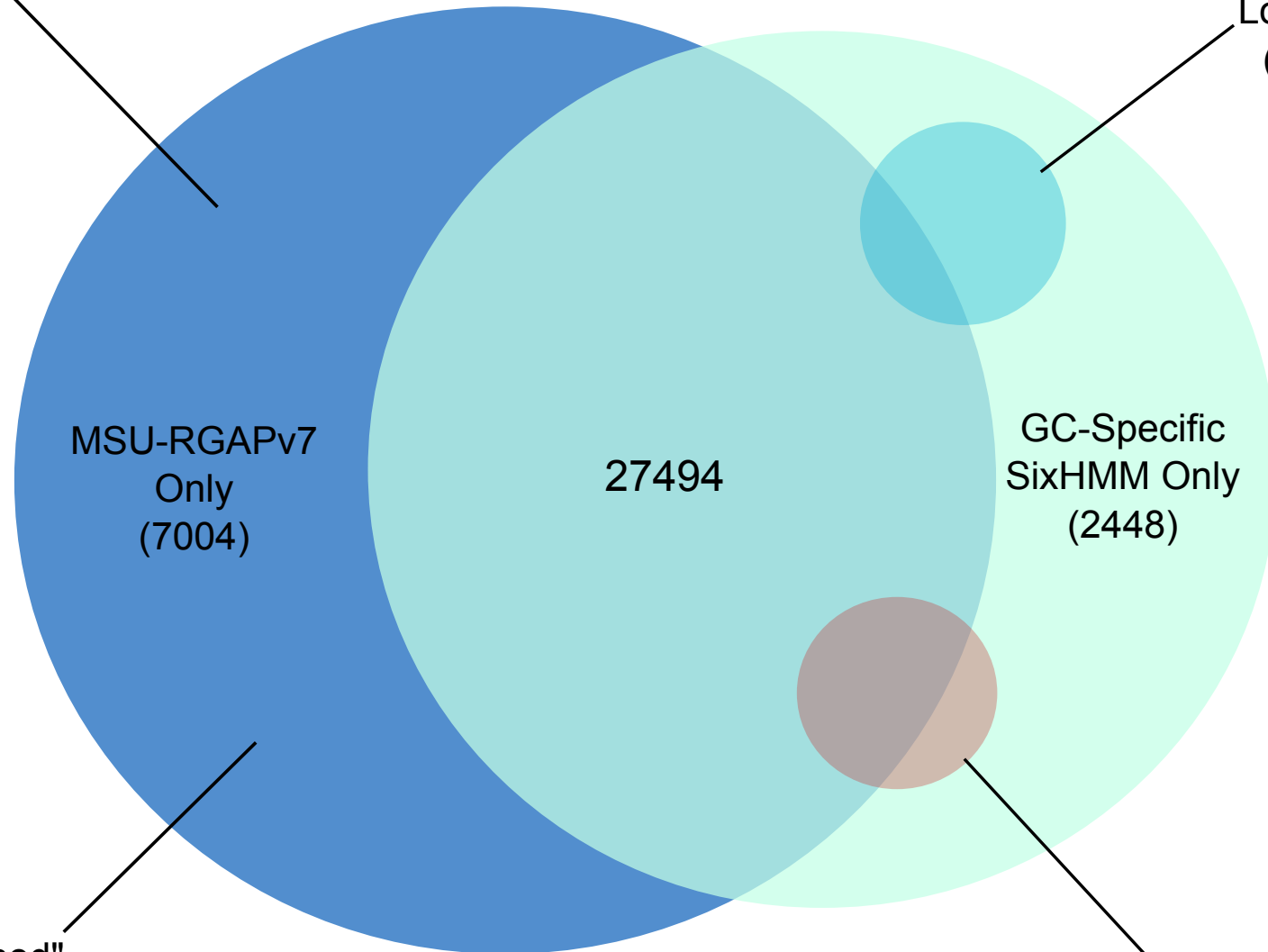

"Expressed"  
Genes  
(4635)

GC-Specific Novel  
High GC  
(282)

Supplement: Supplementary file 1 — Venn diagram depicting the overlap between the rice GC-specific sixHMM annotation and IGRSP v7 annotation. Of the 7004 genes that are only present in the IGRSPv7 annotation, 1365 (19.5%) are designated as “hypothetical”, while 4327 (61.8%) are designated as “expressed”. (PDF 27 kb) [file 12859_2017_1942_MOESM1_ESM.pdf]

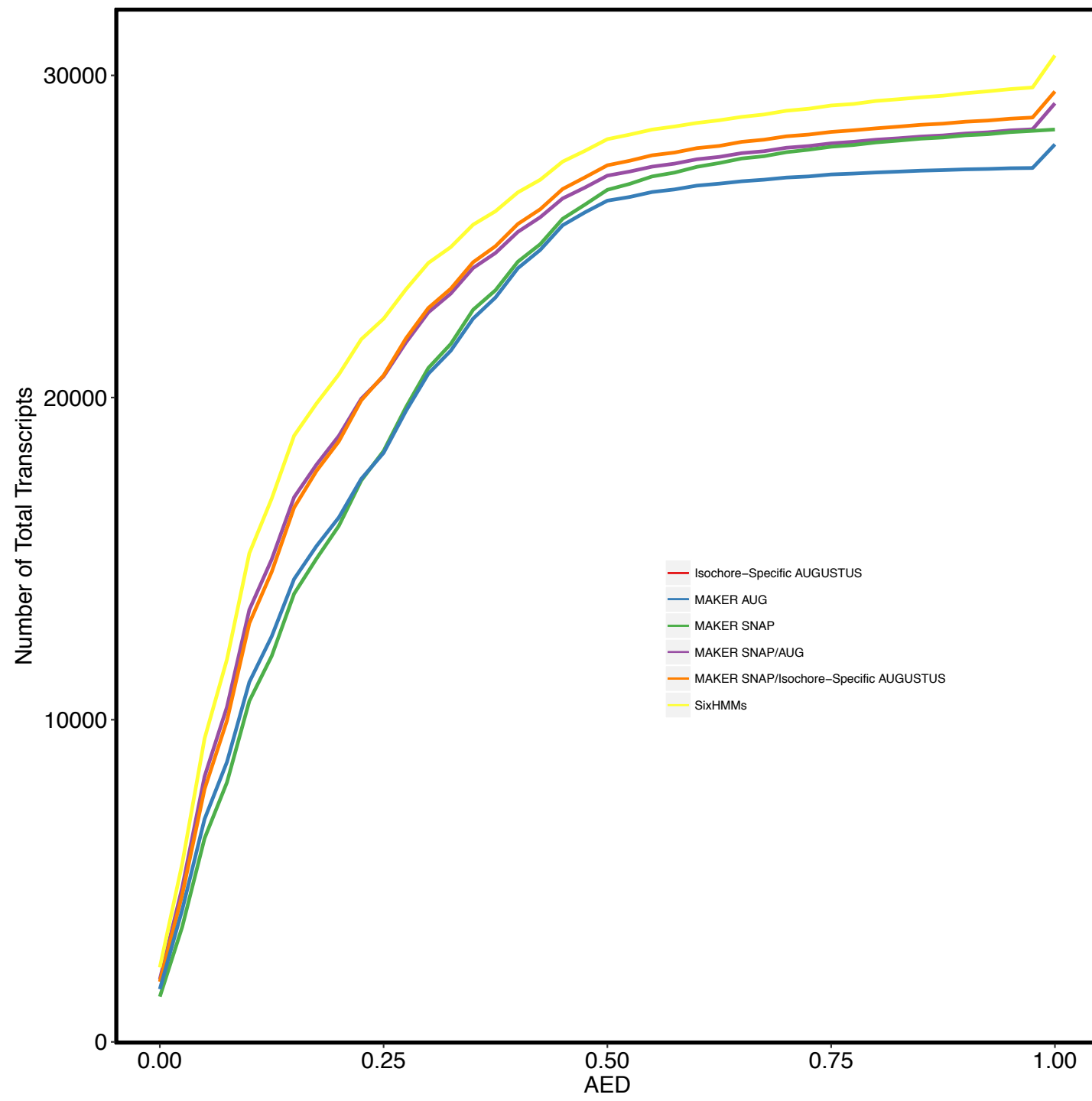

Figure S1

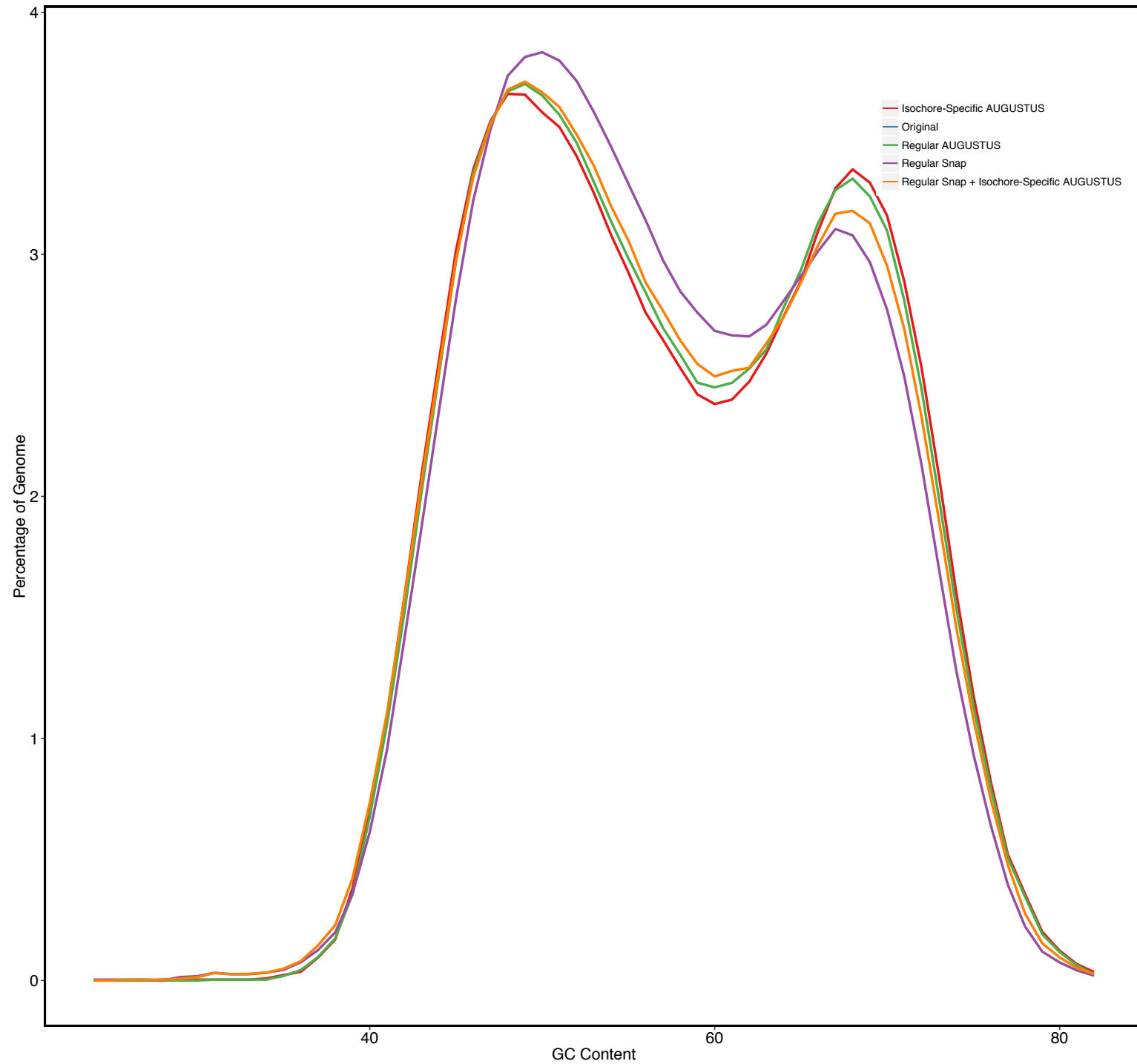

Figure S2

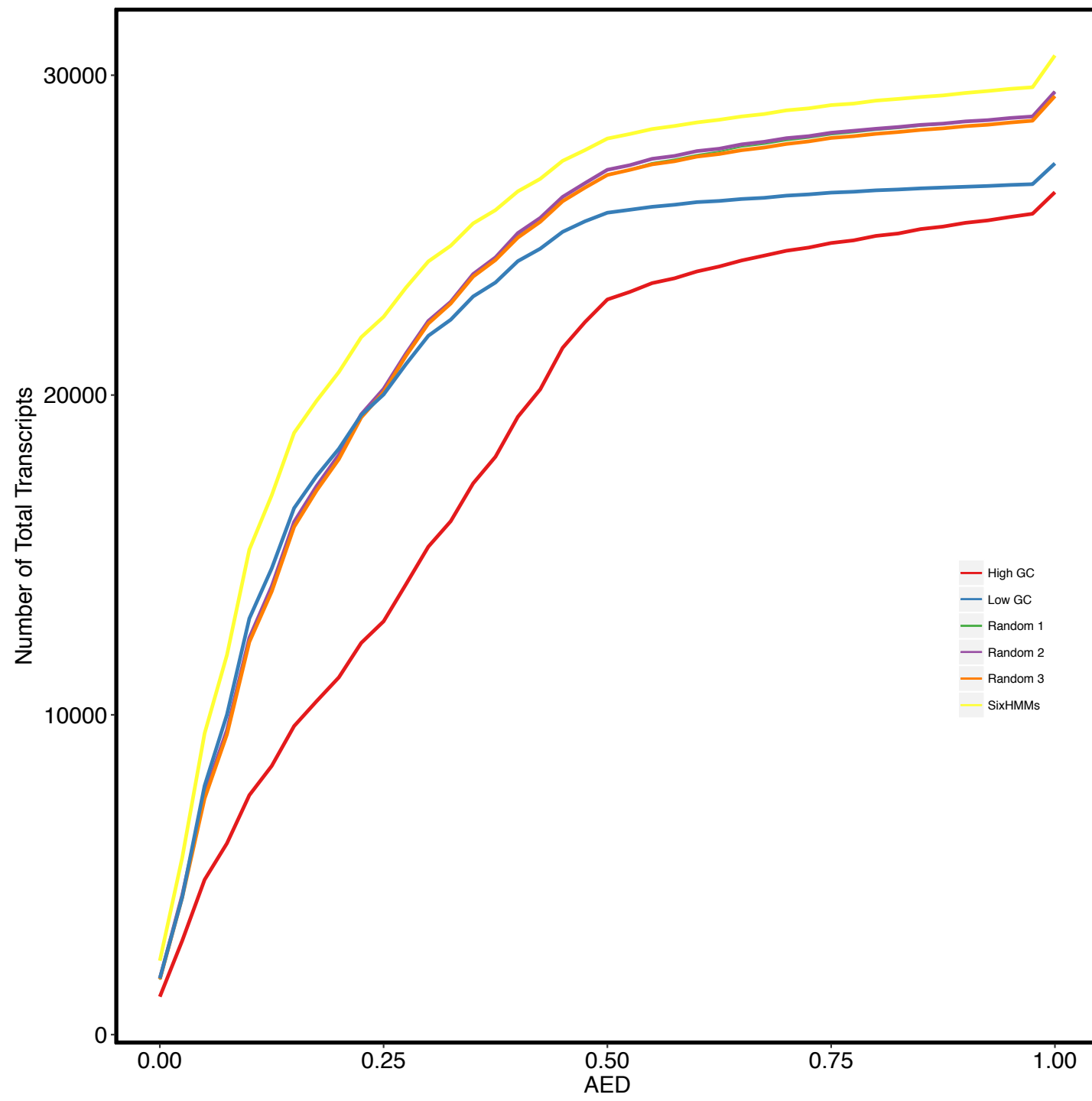

Figure S3

Supplement: Supplementary file 3 — AED curves from various MAKER annotation methods. Figure S1. AED curves of MAKER annotations of Oryza sativa using various ab initio prediction methods. Figure S2. Distribution of GC content of MAKER annotations of Oryza sativa using various ab initio prediction methods. Figure S3. AED curves of MAKER annotations of Oryza sativa using HMMs trained with randomized training data. (PDF 56 kb) [file 12859_2017_1942_MOESM3_ESM.pdf]

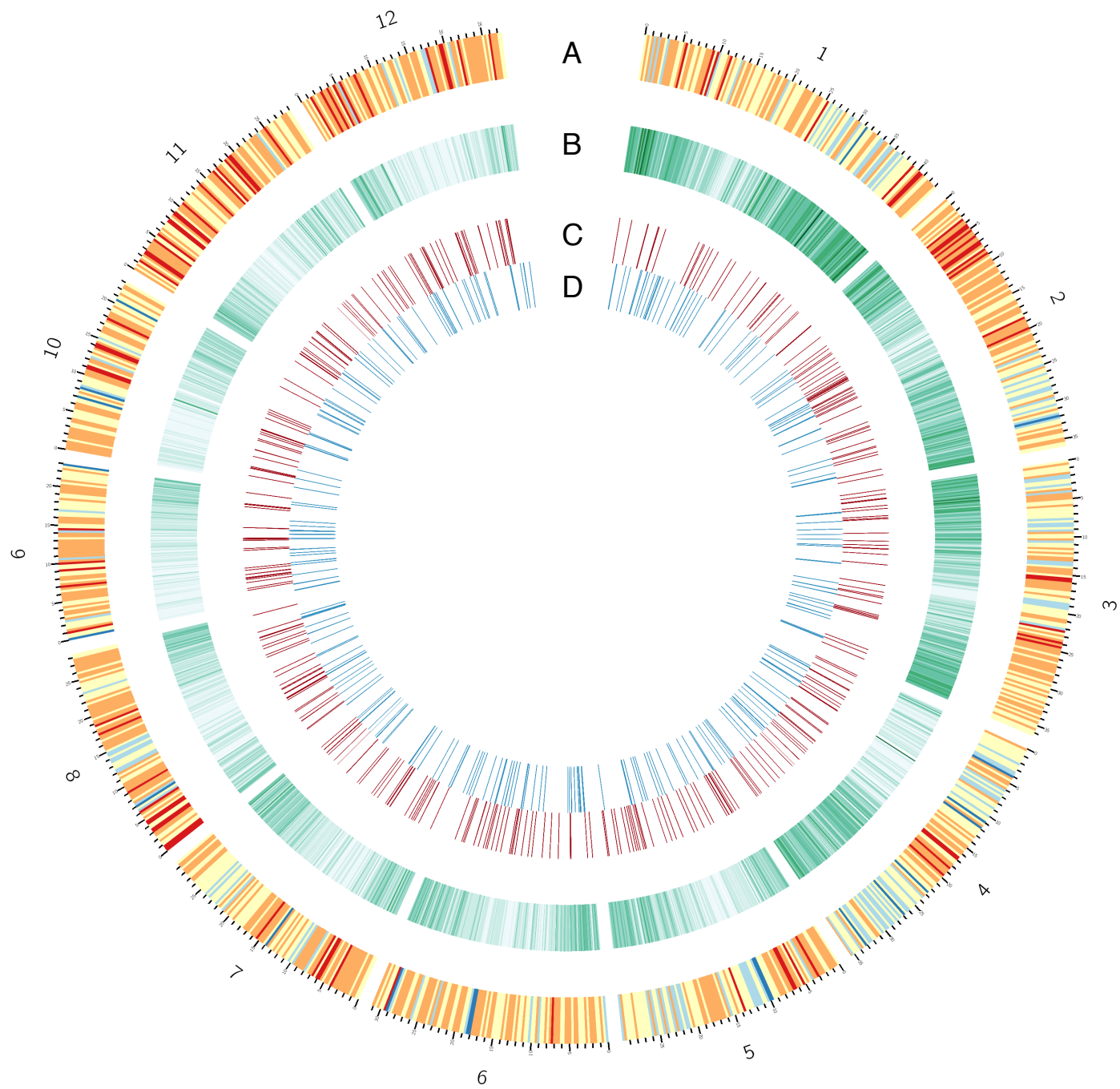

Supplement: Supplementary file 4 — Distribution of GC content, MAKER six HMMs gene predictions and novel genes predicted by the high and low GC HMMs in the Oryza sativa genome. A) Genomic GC content in 300 Mb bins. Warmer colors indicate higher than average GC content while cooler colors indicate lower than average GC content. B) Heatmap visualization of the density of MAKER six HMMs gene models. C) Genomic location of novel genes predicted by the high GC HMMs. D) Genomic location of novel genes predicted by the low GC HMMs. (PDF 1342 kb) [file 12859_2017_1942_MOESM4_ESM.pdf]

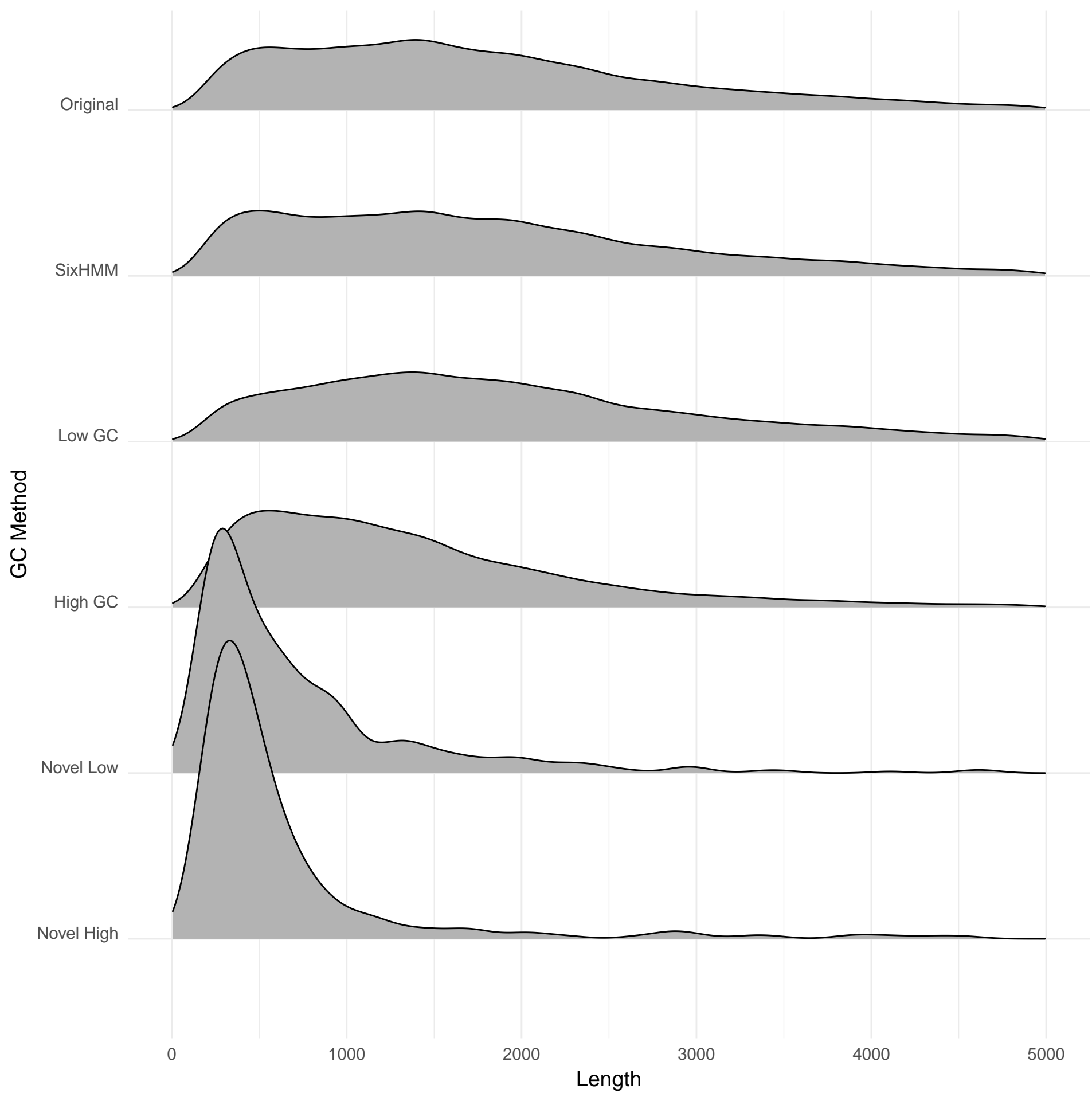

Supplement: Supplementary file 5 — Gene length distributions of MAKER original, six HMMs, low GC, high GC, novel low and novel high GC predictions. Distribution of gene lengths from each of the MAKER annotation methods and from the novel low and high GC gene sets. (PDF 27 kb) [file 12859_2017_1942_MOESM5_ESM.pdf]

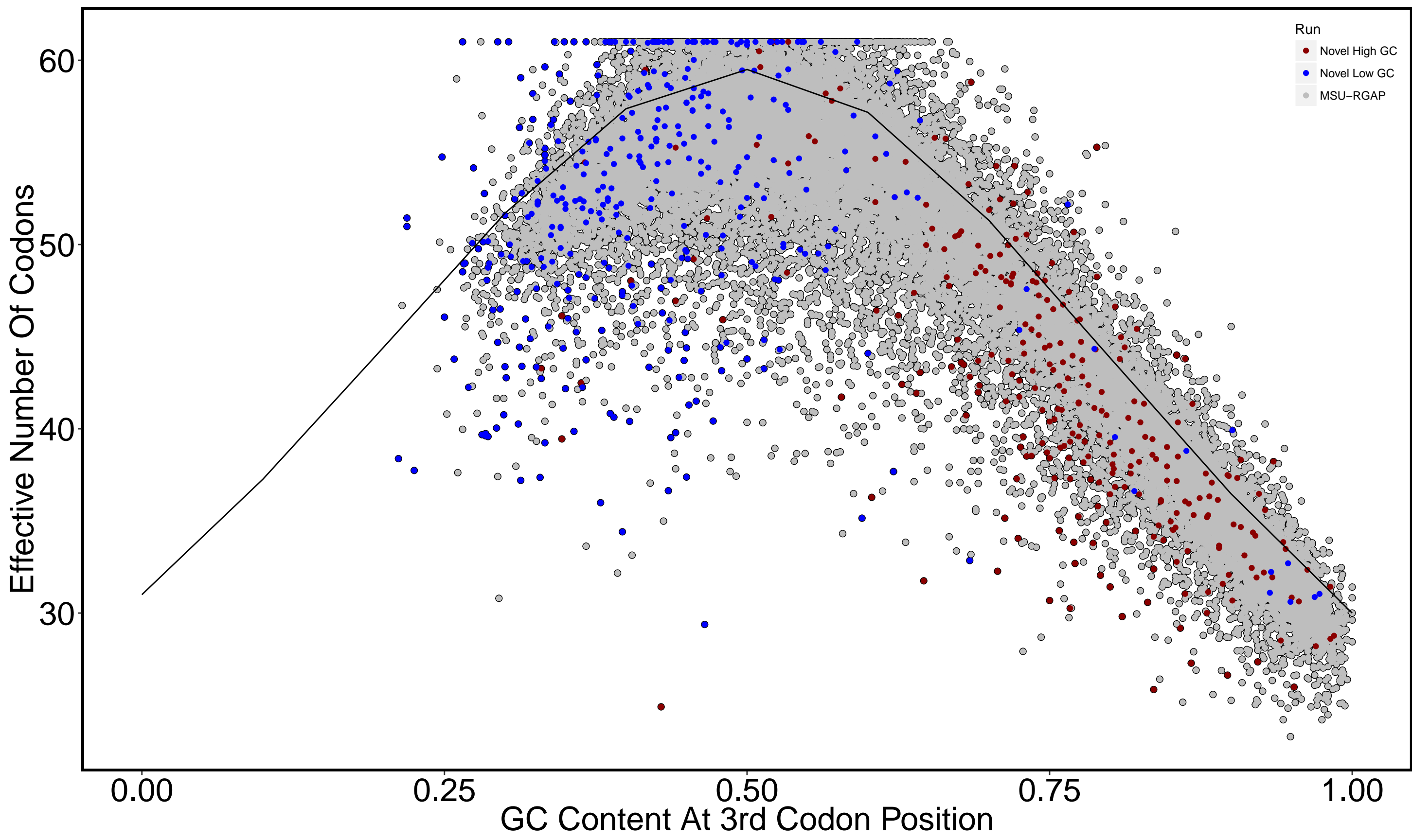

Supplement: Supplementary file 6 — Codon usage of novel high and low GC genes compared to MSU-RGAP annotation. The solid line represents the expected number of codons (Nc) values under a null model where there is no selection on codon usage. (PDF 3224 kb) [file 12859_2017_1942_MOESM6_ESM.pdf]

A

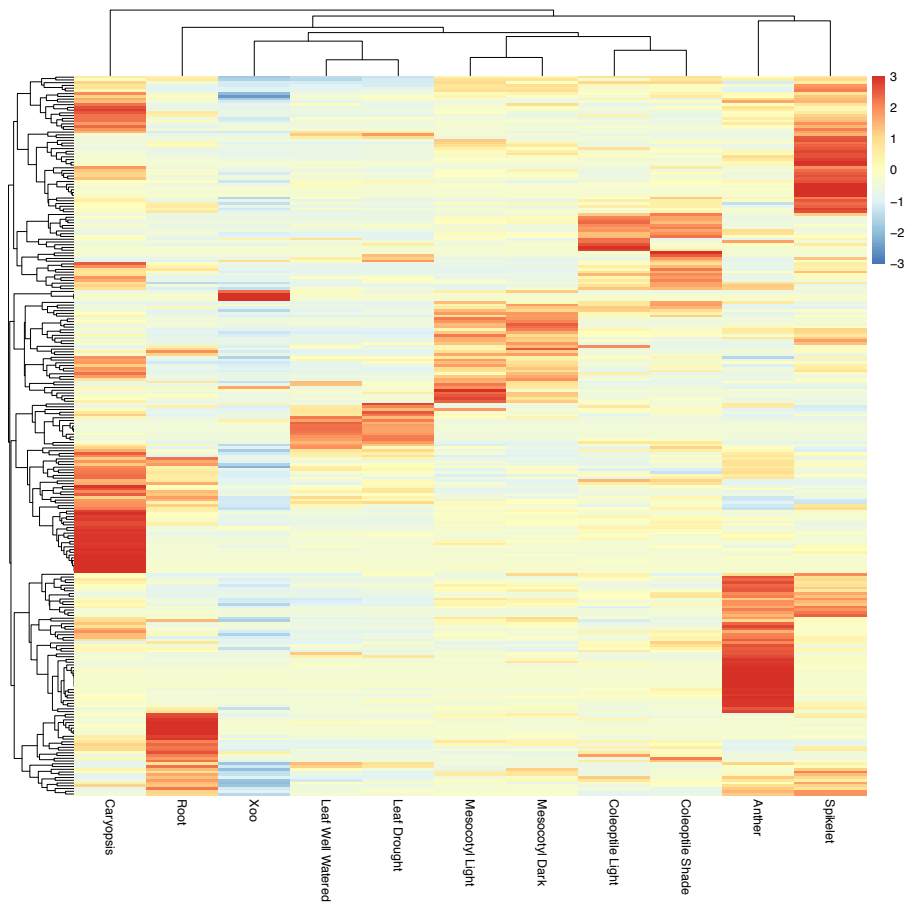

B

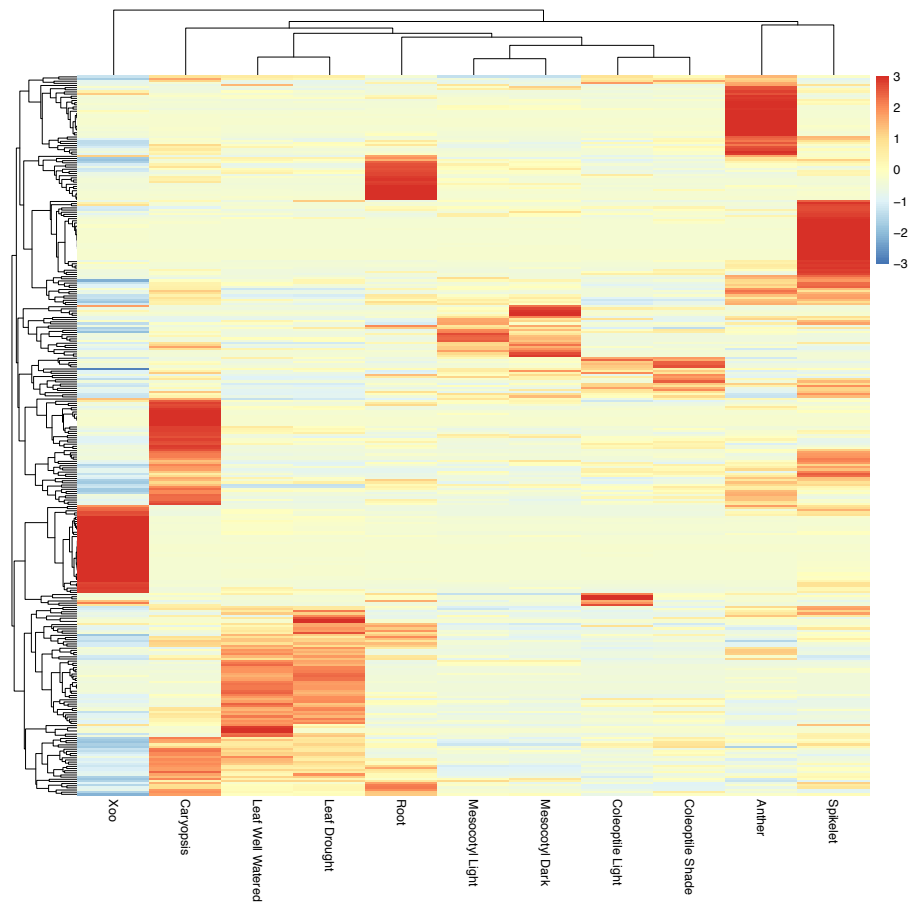

Supplement: Supplementary file 8 — RNA-sequencing analysis of novel high and low GC genes. Heatmap of transcripts per million (TPM) of A) novel genes predicted by low GC HMMs and B) novel genes predicted by high GC HMMs. RNA-sequencing data used for the TPM calculations were obtained from caryopsis, root, leaves inoculated with Xanthomonas oryzae pv. Oryzae, well watered leaves, drought stressed leaves, mesocotyl under light and dark conditions, coleoptile under light and shade conditions, anther and spikelet tissues. Values are scaled by row to a sum of one for visualization purposes. (PDF 128 kb) [file 12859_2017_1942_MOESM8_ESM.pdf]
